# Supplementary material for: Extreme Mobility Creates Contrasting Patterns of Panmixia, Isolation‐by‐Distance and Hybridization in Four Flying‐Fox Species (Pteropus)
Source: Evol Appl. 2026 Oct 15;19(10):e70329. doi: 10.1111/eva.70329 (PMC13673128; doi:10.1111/eva.70329)
Supplement: Supplementary file 1 [file EVA-19-e70329-s002.docx]

# **Supplementary text and figures:** **Extreme mobility creates contrasting patterns of panmixia, isolation-by-distance and hybridization in four flying-fox species (*Pteropus*)**

Armin Scheben^1*^, Adam McKeown^2^, Tom Walsh^3^, David A. Westcott^4^, Suzanne S. Metcalfe^5^, Eric P. Vanderduys^5^ and Bruce L. Webber^6*^

^1^ Simons Center for Quantitative Biology, Cold Spring Harbor Laboratory, Cold Spring

Harbor, NY, 11724, United States of America

^2^CSIRO Environment, Adelaide, South Australia, 5052, Australia

^3^CSIRO Environment, Canberra, Australian Capital Territory, 2600, Australia

^4^CSIRO Environment, Atherton, Queensland, 4883, Australia; Present address: PO Box 1056, Atherton, Queensland, 4883, Australia

^5^CSIRO Environment, Brisbane, Queensland, 4102, Australia

^6^CSIRO Health and Biosecurity, Floreat, Western Australia 6014, Australia; School of Biological Sciences, University of Western Australia, Crawley, Western Australia 6009, Australia; Present Address: Bush Heritage Australia, 637 Flinders St, Docklands, Victoria 3008, Australia.

*To whom correspondence should be addressed: scheben@cshl.edu, bruce.webber@uwa.edu.au

# **Supplementary text**

**Text S1**. **DNA extraction and double digest restriction-site-associated DNA sequencing (ddRAD) library preparation**

Samples were stored in 80% ethanol at room temperature until processing. Genomic DNA was extracted using the Qiagen DNA Blood and Tissue Kit (Hilden, Germany) with an overnight incubation at 56°C until the tissue was completely lysed. The ddRAD libraries (Peterson et al., 2012) were prepared by digesting a total amount of 100 ng of genomic DNA from each individual in a 50 µL reaction with 20 units each of high-fidelity restriction enzymes, *PstI* and *EcoRI* (New England Biolabs, Ipswich MA, USA), 10× CutSmart buffer and water for 30 min at 37°C, followed by a heat inactivation step for 20 min at 65°C. The digestion products were allowed to slowly cool to room temperature and held at 4°C. The digestion product was ligated to the modified P1 and P2 adapters ensuring that each individual had a unique combination of P1 and P2 barcodes. This way twenty-four P1 and four P2 adapters allowed for multiplexing 96 individuals. The ligation reactions were set up in 70 µL volume using the full volume of digestion product with 4 µL of 50 nM P1 and 12 µL of 50 nM P2 adapters, 2000 units of T4 DNA ligase (New England Biolabs, Ipswich MA, USA), 0.15 mM rATP (Promega, Madison, WI, USA), and water and were incubated for 30 min at 22°C. Ligations were heat inactivated for 20 min at 65°C and allowed to slowly cool to room temperature and held at 4°C. Ten µL of adapter-ligated DNA fragments from all individuals were pooled, with careful mixing after each sample addition. The pooling was replicated in three tubes and the following size-selection steps performed on each tube.

The larger fragments (>300 bp) were extracted with 0.65× volume of Agencourt® AMPure® XP magnetic bead solution (Beckman Coulter, Brea, CA, USA) and the bead discarded. The eluent was transferred to a new tube, cleaned with 0.1× volume of bead solution, washed twice with 200 µL of freshly made 80% ethanol, air-dried and resuspended in 20 µL elution buffer (Qiagen, Hilden, Germany). The three replicates were then pooled and well mixed. Finally, 15 µL of size selected DNA was used as a template in a 60 µL PCR reaction with 30 µL of Phusion High Fidelity 2× Master Mix (New England Biolabs, Ipswich MA, USA), 3 µL of 10 µM P1 (AATGATACGGCGACCACCGAGATCTACACTCTTTCCCTACACGACG) and P2 (CAAGCAGAAGACGGCATACGAGATCGTGATGTGACTGGAGTTCAGACGTGTGC) primers and water. PCR conditions were: 98°C for 30 s, 20 cycles of 98°C for 10 s, 60°C for 30 s, 72°C for 40 s, and the final elongation at 72°C for 5 min. The PCR product was cleaned with a 1.8× bead solution and eluted in 40 µL elution buffer to make the final library.

**Text S2. Sample quality control**

A total of 17 samples failed alignment quality control thresholds requiring a >90% paired alignment rate and >800,000 aligned reads. A single sample was excluded based on genotype missingness >55%. A further 6 samples were flagged for expert review and excluded as contaminants based on discordance of preliminary species assignment with sample metadata including sampling location and museum specimen review. For 22 duplicate samples, the sample with the best alignment metrics was selected for further analysis. All samples and relevant exclusion criteria per sample are listed in Table S1. Two *P. poliocephalus* samples collected outside of the usual range of this species in Ingham (samples 1815076 and 1815091) were confirmed to be classified correctly.

**Text S3. Bayesian divergence time estimation**

To investigate between-species divergence-times and validate parameters inferred with fastsimcoal2, we used BEAST2 2.6.7 (Bouckaert et al., 2019) with the plugin SNAPP to infer divergence-times based on SNPs (Stange et al., 2018). We selected three samples without inferred admixture per species retaining a total of 10,394 variable sites. Ascertainment bias correction to adjust parameters based only on variable sites was used. A lognormal prior (real mean=3.54, sd=0.3, offset=0) on the crown group containing *P*. *poliocephalus*, *P. alecto* and *P. conspicillatus* was set to calibrate the analysis based on a previously inferred age of 3.54 mya (Almeida et al., 2014). The moderately high standard deviation was selected to account for the uncertainty inherent in secondary calibrations and to ensure overlap with other slightly deviating estimates for the group (Tsang et al., 2019). The previously inferred maximum likelihood tree was used as a fixed topology to constrain the SNAPP analysis. Inference was run for over 40 million MCMC generations with a 10% burn-in and convergence was confirmed using visual inspection in Tracer 1.7.2 with a criterion of effective sample size (ESS) >200 for each parameter.

# **Supplementary figures**

**Figure S1**. Roost locations and their assignment to distinct geographic regions sampled in this study. To highlight the regions relevant to this study, sampling locations here are shown only for the most widely distributed species *Pteropus* *alecto* and *P*. *conspicillatus*.

**Figure S2**. Comparison of flying-fox DNA degradation based on cytosine deamination between extant samples from CSIRO (n=66) and historical samples from the WA museum (n=15).

**Figure S3**. Genome-wide heterozygosity estimates for A) four flying-fox species and B) six regional populations of *Pteropus alecto* and *P. conspicillatus*. A total of 2,024,709 sites were used for *Pteropus alecto*, 2,518,218 for *P. conspicillatus*, 2,183,168 for *P*. *alecto alecto*, 2,610,770 for *P*. *poliocephalus*, and 2,409,166 for *P*. *scapulatus*.

**Figure S4**. Genome-wide nucleotide diversity (𝜋) estimates for A) four flying-fox species and B) six regional populations of *Pteropus alecto* and *P. conspicillatus*. Means are shown as triangles and specific mean values are shown above each boxplot. The number of sites used for each species is the same as listed in the Figure S3 legend.

**Figure S5**. Genetic differentiation between four flying-fox species based on F_ST_.

**Figure S6**. Genetic differentiation between geographic populations of *P*. *alecto* and *P*. *conspicillatus* based on F_ST_. The Wet Tropics Australia and New Guinea groups represent *P*. *conspicillatus* and the other groups represent *P. alecto*.

**Figure S7.** SNP-based principal component analysis (PCA) of *P. alecto* using the “alecto + conspicillatus” dataset colored by A) geographic populations and B) genetic populations.

**Figure S8**. SNP-based principal component analysis (PCA) of flying-fox populations across three genotype missingness thresholds. For the species-level PCA, a genotype missingness threshold of 50% (22,394 SNPs) is shown in A), 40% (19,501 SNPs) in C), and 30% (16,160 SNPs) in E). For the population-level PCA of *P*. *alecto* and *P*. *conspicillatus*, a genotype missingness threshold of 50% (33,830 SNPs) is shown in B), 40% (29,222 SNPs) in D), and 30% (23,946 SNPs) in F).

**Figure S9**. SNP-based principal component analysis (PCA) of *P*. *alecto* and *P*. *conspicillatus* populations using 216 SNPs with no missing genotypes.

**Figure S10**. Triangle plot of admixture between *P*. *alecto* and *P*. *conspicillatus* individuals based on 15 ancestry-informative SNP markers. The hybrid index of F_1_ and F_2_ hybrids is expected to be 0.5 while backcross (BC) individuals between hybrids and parental lineages are expected to lie on the edges of the triangle with elevated heterozygosity and hybrid indices. Outliers are labelled with sample IDs in boxes. Proportions of missing SNP markers were not associated with high interclass heterozygosity or hybrid indices (Figure S11).

**Figure S11**. Triangle plot of admixture between *P*. *alecto* and *P*. *conspicillatus* individuals based on 15 ancestry-informative SNP markers colored by the proportion of missing markers per individual. Outliers are labelled with sample IDs in boxes.

**Figure S12**. SNP-based maximum likelihood phylogeny of *Pteropus* *alecto* and *P*. *conspicillatus*. A total of 17,708 SNP sites were used. Individuals are highlighted in a color by geographic region and the Indonesian *P. alecto alecto* group is used as the outgroup. Historical *P. alecto* samples were collected from 1989 to 2003 (see Table S1). Bootstrap support values >70 are shown.

**Figure S13**. SNP-based maximum likelihood phylogeny of *Pteropus* *poliocephalus* and *P*. *scapulatus*. A total of 11,818 SNP sites were used. Individuals are highlighted in a color by geographic region. Bootstrap support values >70 are shown, highlighting robustly supported branches.

**Figure S14**. SNP-based phylogeny of *Pteropus* *alecto* and *P*. *conspicillatus* inferred by CASTER-site. A total of 17,708 SNP sites were used. Individuals are highlighted in a color by geographic region. Branch bootstrap support values >95 are shown.

**Figure S15**. SNP-based phylogeny of *Pteropus* *poliocephalus* and *P*. *scapulatus* inferred by CASTER-site. A total of 11,818 SNP sites were used. Individuals are highlighted in a color by geographic region. Branch bootstrap support values >95 are shown.

**Figure S16**. Gene flow in *Pteropus alecto* and *P. conspicillatus* along a geographic ring ranging across Indonesia, Australia and Papua New Guinea. A) Maximum likelihood tree with migration edges for three genetic *P. alecto* populations and *P. conspicillatus*. The diverged taxon *P. alecto alecto* was used as the outgroup. B) Map of inferred migration scenarios between populations using coalescent simulations (see Table 1 for additional inferred parameters). The following population codes were used in A) and B): INDO+3NWA, Indonesia and three samples from North West Australia; NWA+BURK, remaining North West Australian samples and all Burketown samples; EA+NEA, East Australia and North East Australia; WTA+NG, Wet Tropics Australia and New Guinea.

**Figure S17**. Comparison of the observed and simulated joint 2D site frequency spectrum (SFS) under the best-fit demographic model across flying-fox population pairs (A-E). Log-transformed counts of sites are shown for minor allele frequency (MAF) pairs. Monomorphic sites are omitted. The following population codes were used: INDO+3NWA, Indonesia and three samples from North West Australia; NWA+BURK, remaining North West Australian samples and all Burketown samples; EA+NEA, East Australia and North East Australia; WTA+NG, Wet Tropics Australia and New Guinea.

**Figure S18.** Comparison of the observed and simulated joint 2D site frequency spectrum (SFS) under the best-fit demographic model for Australian and Indonesian *Pteropus alecto*. The SFS was computed using A) all samples and B) excluding the samples from North West Australia genetically clustered with the Indonesian population (samples M53754, M54659, and M54660). Log-transformed counts of sites are shown for minor allele frequency (MAF) pairs. Monomorphic sites are omitted. The following population codes were used: INDO+3NWA, Indonesia and three samples from North West Australia; INDO, Indonesia; NWA+EA+NEA, North West Australia, East Australia and North East Australia.

**Figure S19**. Dated phylogeny of sampled flying-fox species inferred using BEAST and SNAPP from genome-wide SNPs for three samples per branch. Mean divergence estimates per node are ages in millions of years before present. Blue bars show the 95% highest posterior density interval for each node. The subspecies designation *Pteropus alecto gouldi*, used to describe Australian *P. alecto*, is used here to indicate that the three sampled individuals for this branch all originate from Australia.

**Supplementary references**

Almeida, F. C., Giannini, N. P., Simmons, N. B., & Helgen, K. M. (2014). Each flying fox on its own branch: A phylogenetic tree for *Pteropus* and related genera (Chiroptera: Pteropodidae). *Molecular Phylogenetics and Evolution*, *77*, 83–95. https://doi.org/10.1016/j.ympev.2014.03.009

Bouckaert, R., Vaughan, T. G., Barido-Sottani, J., Duchêne, S., Fourment, M., Gavryushkina, A., Heled, J., Jones, G., Kühnert, D., Maio, N. D., Matschiner, M., Mendes, F. K., Müller, N. F., Ogilvie, H. A., Plessis, L. du, Popinga, A., Rambaut, A., Rasmussen, D., Siveroni, I., … Drummond, A. J. (2019). BEAST 2.5: An advanced software platform for Bayesian evolutionary analysis. *PLoS Computational Biology*, *15*(4), e1006650. https://doi.org/10.1371/journal.pcbi.1006650

Peterson, B. K., Weber, J. N., Kay, E. H., Fisher, H. S., Hoekstra, H. E. (2012) Double digest RADseq: an inexpensive method for de novo SNP discovery and genotyping in model and non-model species. *PLoS One*, *7*(5): e37135. https://doi.org/10.1371/journal.pone.0037135

Stange, M., Sánchez-Villagra, M. R., Salzburger, W., & Matschiner, M. (2018). Bayesian divergence-time estimation with genome-wide single-nucleotide polymorphism data of sea catfishes (Ariidae) supports Miocene closure of the Panamanian isthmus. *Systematic Biology*, *67*(4), 681–699. https://doi.org/10.1093/sysbio/syy006
